# Urinary reabsorption in the rat kidney by anticholinergics

**DOI:** 10.1038/s41598-021-88738-y

**Published:** 2021-04-28

**Authors:** Hideki Oe, Hatsumi Yoshiki, Xinmin Zha, Hisato Kobayashi, Yoshitaka Aoki, Hideaki Ito, Osamu Yokoyama

**Affiliations:** Department of Urology, Faculty of Medical Science, University of Fukui, 23-3 Matsuokashimoaizuki, Eiheiji-cho, Yoshida-gun, Fukui 910-1193 Japan

**Keywords:** Nephrology, Urology

## Abstract

Anticholinergics, therapeutic agents for overactive bladder, are clinically suggested to reduce urine output. We investigated whether this effect is due to bladder or kidney urine reabsorption. Various solutions were injected into the bladder of urethane-anesthetized SD rats. The absorption rate for 2 h was examined following the intravenous administration of the anticholinergics imidafenacin (IM), atropine (AT), and tolterodine (TO). The bilateral ureter was then canulated and saline was administered to obtain a diuretic state. Anticholinergics or 1-deamino-[8-D-arginine]-vasopressin (dDAVP) were intravenously administered. After the IM and dDAVP administrations, the rat kidneys were immunostained with AQP2 antibody, and intracellular cAMP was measured. The absorption rate was ~ 10% of the saline injected into the bladder and constant even when anticholinergics were administered. The renal urine among peaked 2 h after the saline administration. Each of the anticholinergics significantly suppressed the urine production in a dose-dependent manner, as did dDAVP. IM and dDAVP increased the intracellular cAMP levels and caused the AQP2 molecule to localize to the collecting duct cells' luminal side. The urinary reabsorption mechanism through the bladder epithelium was not activated by anticholinergic administration. Thus, anticholinergics suppress urine production via an increase in urine reabsorption in the kidneys' collecting duct cells via AQP2.

## Introduction

Of the several lower urinary tract symptoms that an individual may experience, nocturia most affects the quality of life and overall health, and among the causes of nocturia, the most difficult factor to treat is nocturnal polyuria. More than 76% of patients who go to the toilet twice or more at night have nocturnal polyuria^1,2^. Anticholinergics, when administered before sleeping, have been suggested to reduce the nocturnal urine volume^3,4^. In basic research to test that suggestion, Watanabe et al. confirmed that the anticholinergics IM and TO administered separately to a rat diuretic model reduced the urine volume^5^. However, the mechanism by which anticholinergics reduce urine output has not been elucidated.

Muscarinic acetylcholine (ACh) receptors (mAChRs) are present in the murine renal collecting ducts^6^. The mAChR agonist carbachol activates protein kinase C (PKC) via an upregulation of the phosphoinositide pathway, and its application resulted in the inhibition of the water permeability of rat terminal inner medullary collecting ducts^7^. ACh works on diuresis by promoting a PKC-mediated pathway, and anticholinergics may thus exert antidiuretic effects by inhibiting this signal. On the other hand, the rat urinary bladder absorbs water and salts under the full-filled condition^8^. Aquaporin-2 (AQP2) was suggested to be involved in urine absorption from the rat bladder^8^.

As a member of the AQP family, AQP2 plays an important role in regulating the body's water balance. AQP2 is present mainly in the collecting ducts' principal cells of the kidney^9^. AQP2 in the renal collecting ducts is regulated by the anterior pituitary hormone arginine vasopressin (AVP). AVP binds to the basolateral G-protein-coupled type 2 vasopressin receptor and stimulates the exocytosis of AQP2 water channels in the apical membrane of the renal collecting ducts (AQP2 trafficking) via a cAMP-protein kinase A pathway, resulting in increasing water reabsorption^10^. This response is completed within 5–30 min after the increase in the plasma AVP concentration. AQP2 traffics between the intracellular membrane compartment and the apical plasma membrane by exocytotic membrane insertion and endocytotic internalization^11^.

With this background, we conducted animal experiments to investigate the following mechanisms that may underlie the reduction of urine output with anticholinergics: (1) the reabsorption of urine in the bladder, and (2) the reabsorption of urine in the kidneys. Our findings elucidate the mechanism of action of anticholinergics as new candidates for reducing the urinary volume in nocturnal polyuria, and we describe the possibility of replacing desmopressin or reducing its side effects.

## Results

### Reabsorption in the bladder

#### Changes in the amounts of the bladder solutions, electrolytes, and osmolality

**Figure 1:**
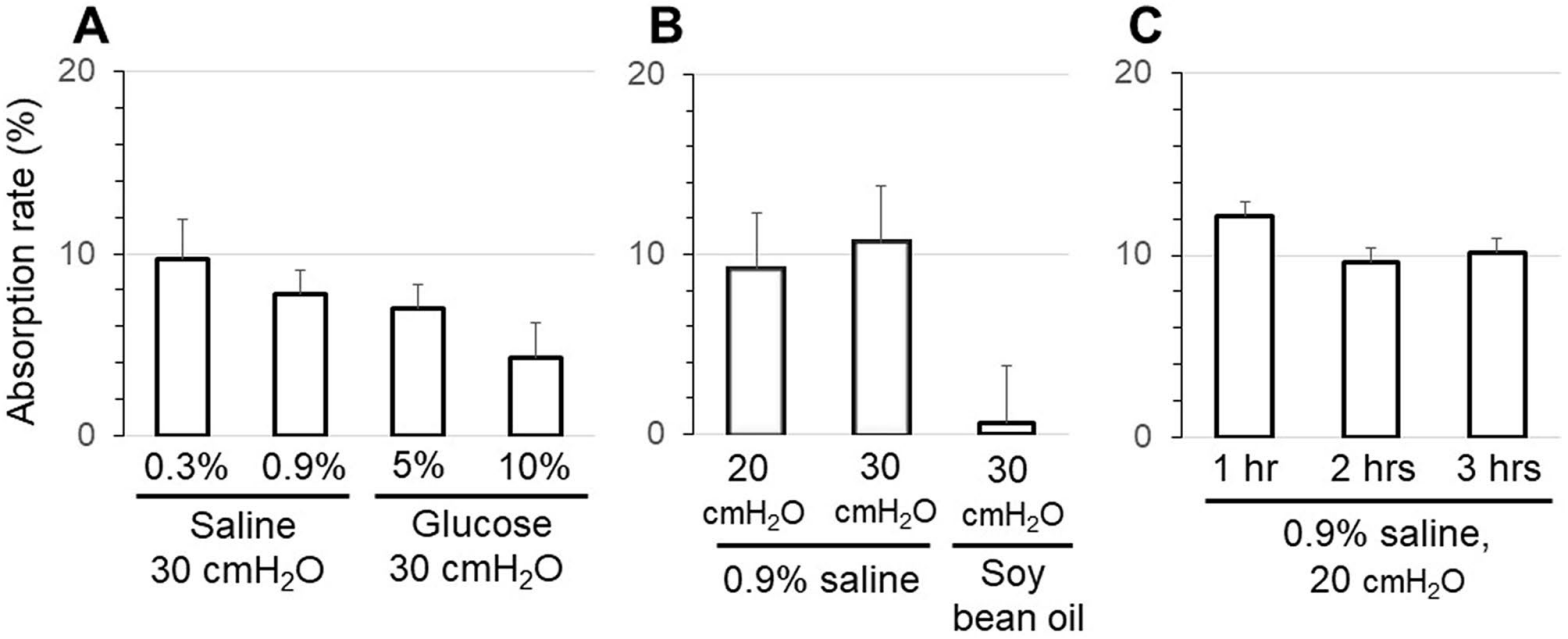
(**A**) The absorption rate of the rat bladder wall was compared under various conditions. Comparison between 0.3 and 0.9% saline and between 5 and 10% glucose under 30 cmH_2_O pressure. (**B**) Comparison when the bladder pressure was maintained at 20 cmH_2_O and 30 cmH_2_O for 2 h and when the bladder was filled with soybean oil. (**C**) Comparison when the bladder pressure was maintained at 20 cmH_2_O for 1, 2, or 3 h. Values are mean ± SEM of measurements in 8 rats.

Physiological saline injected into the rat bladder was absorbed from the bladder, and the absorption rate was calculated. Our comparisons of the 0.3% and 0.9% saline under 30 cmH_2_O pressure and of the 5% and 10% glucose solutions (Fig. 1A) revealed that the average absorption rate was ≤ 10% under any of the conditions used. When the bladder pressure was maintained at 20 cmH_2_O or 30 cmH_2_O for 2 h, the average absorption rate was around 10% (Fig. 1B). The absorption rate was markedly lower when the bladder was filled with soybean oil. Even when the bladder pressure was maintained at 20 cmH_2_O for 1, 2, or 3 h, approx. 10% of the injected amount was absorbed (Fig. 1C).

**Figure 2:**
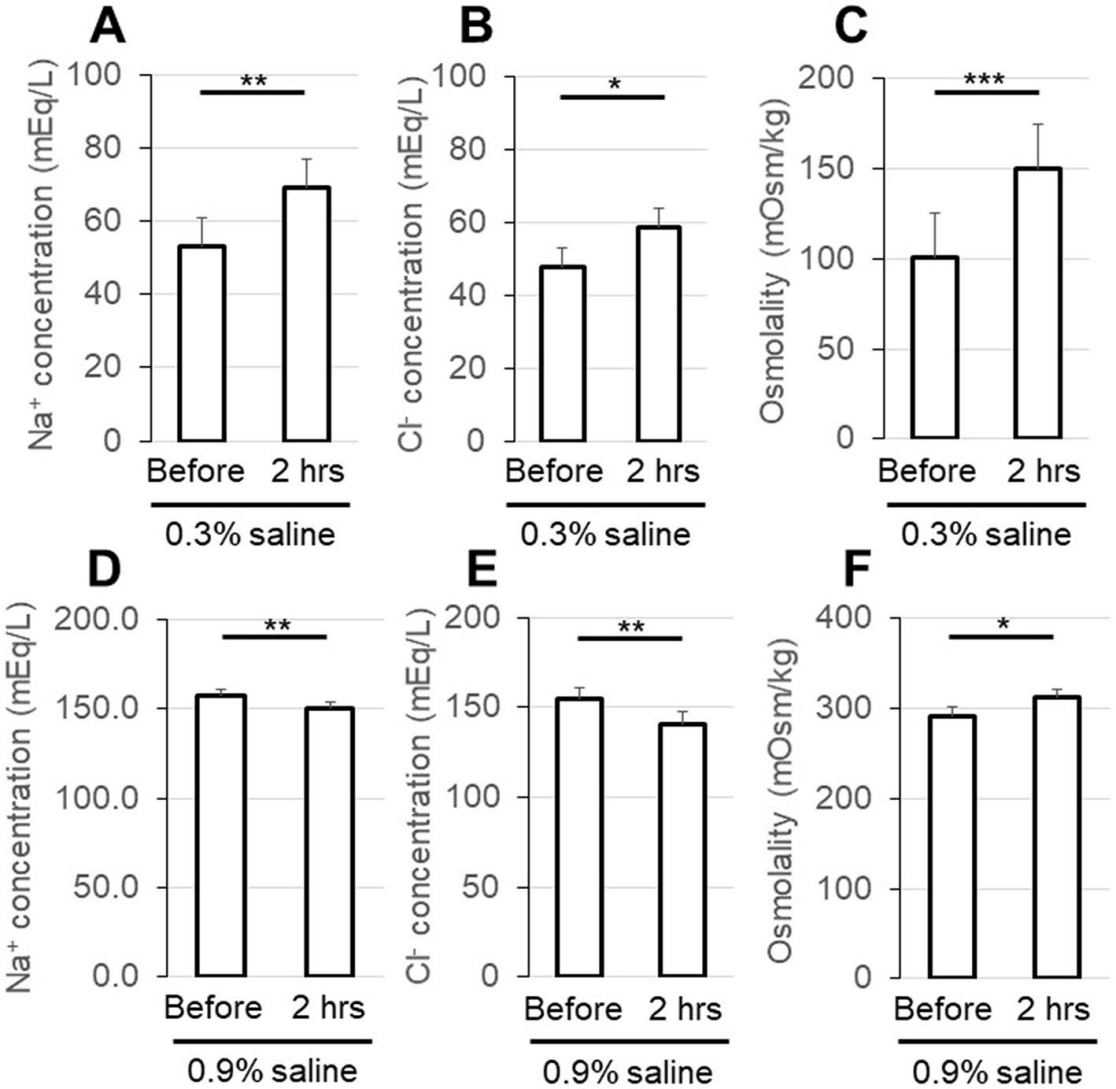
Comparison of electrolytes and osmolality of the injected solution before and 2 h after 0.3% (**A**–**C**) or 0.9% saline (**D**–**F**) was injected into the bladder. Bladder pressure was maintained at 30 cmH_2_O for 2 h. The Na^+^ concentration was significantly increased when 0.3% saline was injected (**A**) but decreased when 0.9% saline was injected (**D**). The Cl^−^ concentration was also significantly increased when 0.3% saline was injected (**B**) and decreased when 0.9% saline was injected (**E**). The osmolality showed similar changes; it significantly increased when 0.3% saline was injected (**C**) and significantly decreased when 0.9% saline was injected (**F**). Values are the mean ± SEM of measurements in 8 rats. *p < 0.05, **p < 0.01, ***p < 0.001 between values.

Electrolytes and osmolality of the injected solution were evaluated before and 2 h after 0.3% or 0.9% saline was injected into the bladder. The Na^+^ concentration was significantly increased when 0.3% saline was injected (Fig. 2A), but it was decreased when 0.9% saline was injected (Fig. 2D). The Cl^−^ concentration was also significantly increased when 0.3% saline was injected (Fig. 2B) and decreased when 0.9% saline was injected (Fig. 2E). Osmolality showed similar changes; it was significantly increased when 0.3% saline was injected (Fig. 2C) and significantly decreased when 0.9% saline was injected (Fig. 2F).

#### Changes in the amounts of the bladder solutions, electrolytes, and osmolality with anticholinergics

**Figure 3:**
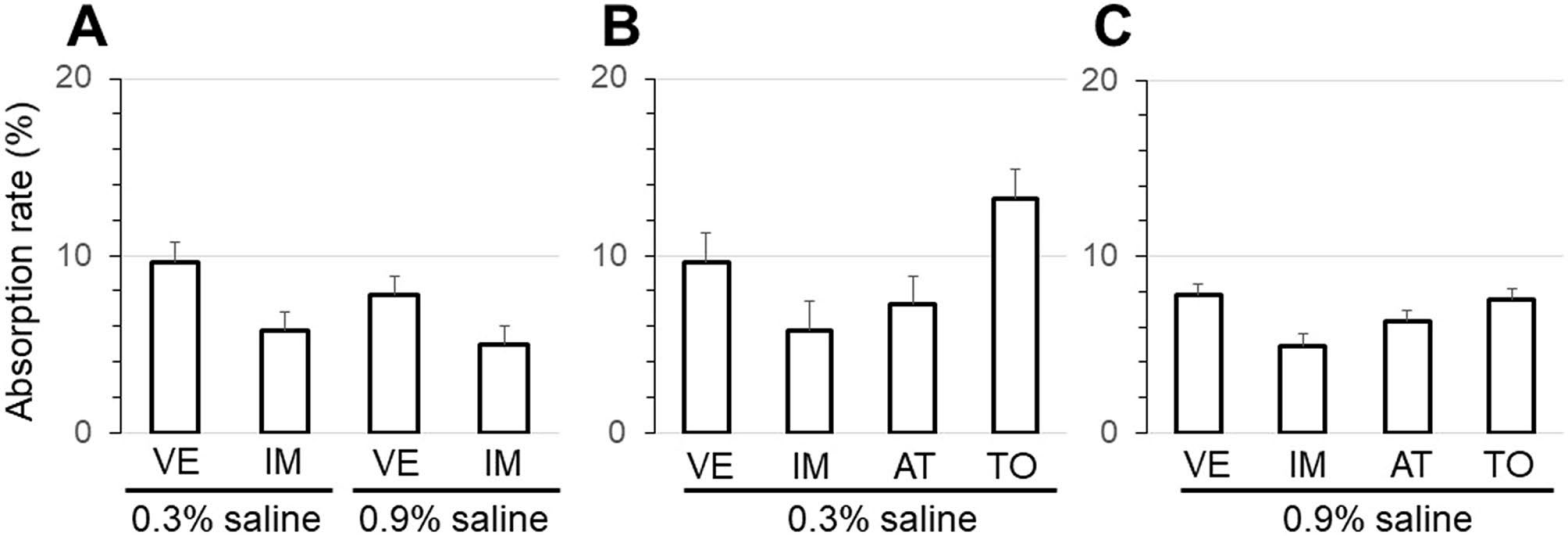
The absorption rate of the rat bladder wall was compared by administering three different anticholinergics. (**A**) Comparison between intravenous vehicle (VE) and imidafenacin (IM) (0.1 mg/kg) administration when the bladder was filled with 0.3% or 0.9% saline. Comparison among intravenous VE, IM (0.1 mg/kg), atropine (AT) (0.1 mg/kg), and tolterodine (TO) (0.3 mg/kg) administration when the bladder was filled with 0.9% saline (**C**). Values are the mean ± SEM of measurements in 8 rats.

When the rat bladder was filled with 0.3% or 0.9% saline, the average absorption rate after the intravenous administration of the anticholinergic IM (0.1 mg/kg) was not significantly different from that after intravenous VE (vehicle) (Fig. 3A). In addition, when the bladder was filled with 0.3% or 0.9% saline, the average absorption rates provided by intravenous IM (0.1 mg/kg), AT (0.1 mg/kg), and TO (0.3 mg/kg) were not significantly different from that by intravenous VE (Fig. 3B,C). Even when the anticholinergics were administered, no significant increase or decrease in electrolytes or osmolality of the injected solution were observed (data not shown).

### Reabsorption in the kidney

#### Changes in the urine amount

**Figure 4:**
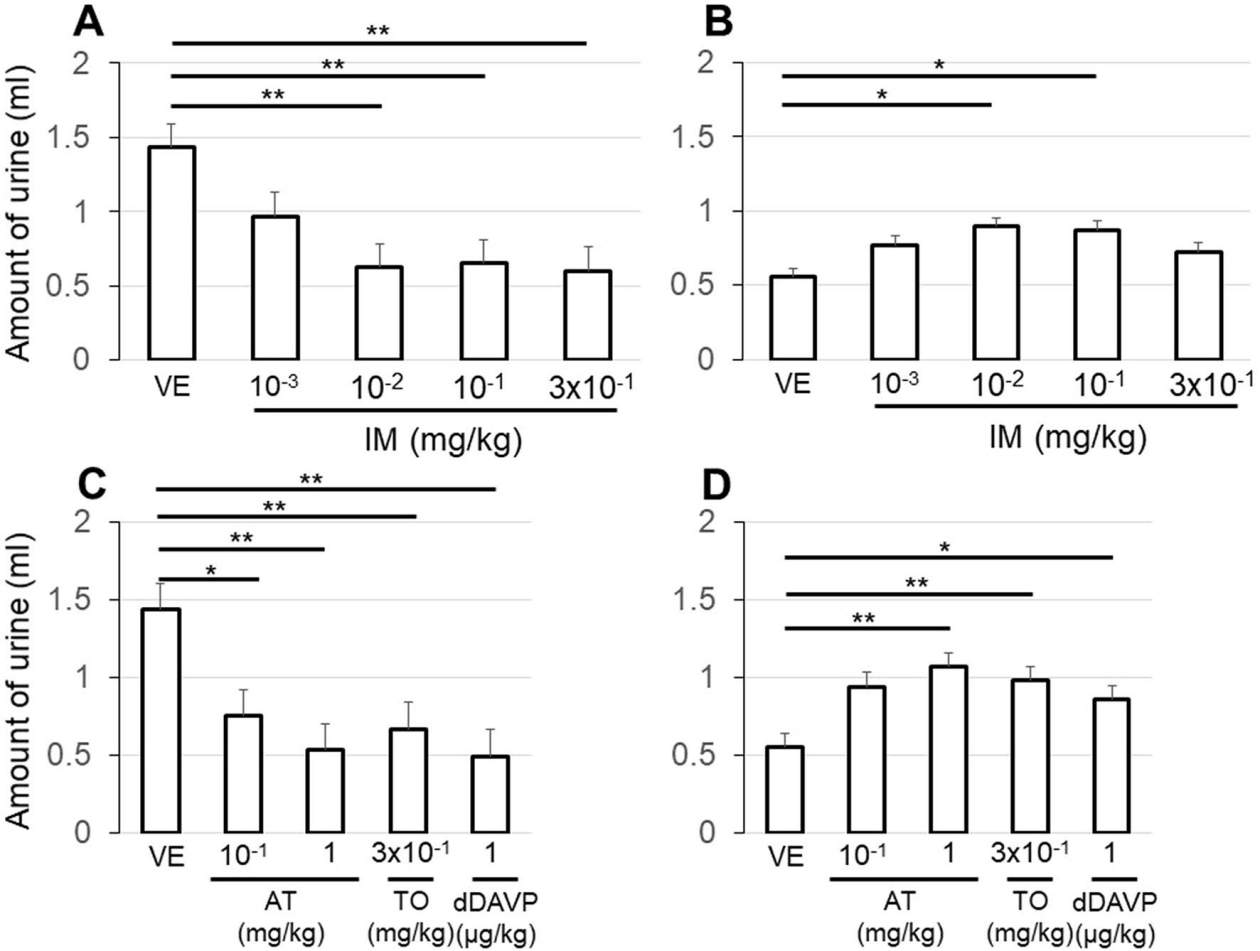
The amount of urine per 2 h from the kidney was compared after the administrations of VE and three anticholinergics. Dose-dependent suppressions of urine production were observed in the first 2 h with the administrations of IM (**A**) and AT (**C**) compared to VE. Urine production was also suppressed by TO and dDAVP (**C**). In the next 2 h, urine production was increased compared to VE (**B**,**D**). Values are the mean ± SEM of measurements in 8 rats. *p < 0.05, **p < 0.01 between values.

The rats' renal urine production peaked 2–4 h after the intravenous administration of saline. We compared the amounts of urine per 2 h after the administration of VE, the three anticholinergics, and dDAVP. The use of IM significantly suppressed the urine production in a dose-dependent manner (p < 0.01 vs. VE) (Fig. 4A). The peak of urine production was also suppressed by the AT, TO, and dDAVP administrations (Fig. 4C). In the 2 h following the peak, the urine production by the rats treated with an anticholinergic or dDAVP was increased, and significant differences were observed compared to VE (Fig. 4B,D).

#### Changes in sodium excretion

**Figure 5:**
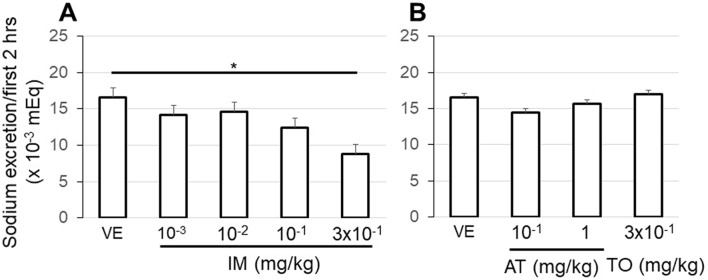
The sodium excretion from the kidney was compared between intravenous VE and anticholinergics administration. Urine was collected for the first 2 h after an intravenous administration of an anticholinergic to measure the amount of sodium in the urine. Comparisons were performed between intravenous VE and IM (10^−3^ , 10^−2^, 10^−1^, 3 × 10^−1^ mg/kg; A) and between VE and AT (10^−1^, 1 mg/kg), TO (3 × 10^−1^ mg/kg). Values are the mean ± SEM of measurements in 8 rats. *p < 0.05 between values.

We calculated the sodium excretion every 2 h and compared the values between VE-administered and IM-administered rats (Fig. 5A,B). No differences were observed in sodium excretion for the first 2 h between the VE and IM (10^−3^ to 10^−1^ mg/kg), AT (10^−1^, 1 mg/kg), and TO (3 × 10^−1^ mg/kg) groups. However, a significant reduction in sodium excretion was recognized in the group that received the high dose of IM (3 × 10^−1^ mg/kg). In the next 2 h, the amount of sodium excretion tended to increase in the anticholinergic groups, but there were no significant differences compared with the VE group (data not shown).

#### Changes in cAMP contents

**Figure 6:**
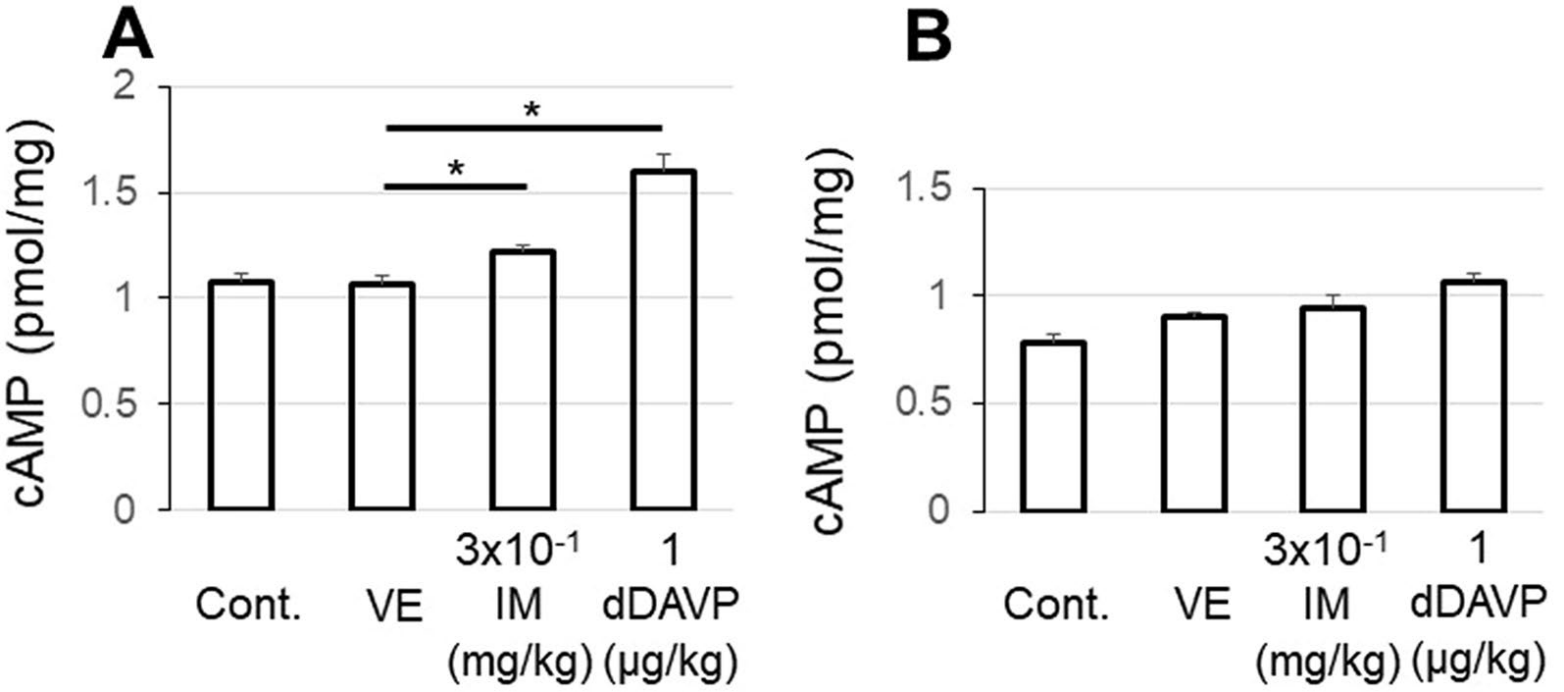
The amount of intracellular cAMP induced by an intravenous administration of VE, IM, or dDAVP in the renal cortex (**A**) and medulla (**B**). The amount of cAMP before saline administration (the non-diuretic state) was adopted as the control. VE, IM, or dDAVP was intravenously injected 2 h after the start of saline administration, and 1 h later, sample kidneys were harvested. Values are the mean ± SEM of measurements in 8 rats. *p < 0.05 between values.

The level of cAMP before the saline administration (the non-diuretic state; control) was the same as that in the diuretic state. The administrations of IM (3 × 10^−1^ mg/kg) and dDAVP (1 µg/kg) significantly increased the cAMP content in the renal cortex compared to VE (Fig. 6A). No significant increase was observed in the medulla (Fig. 6B).

#### Immunolocalization of AQP2 in the rat kidney

**Figure 7:**
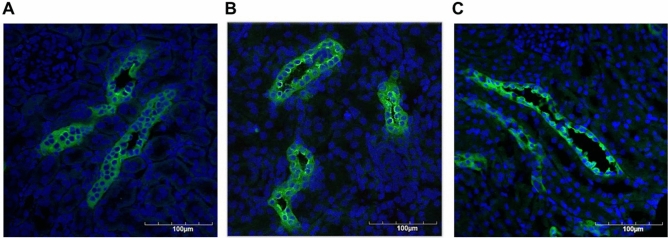
The changes in the distribution of AQP2 in the collecting ducts of the kidney are shown. Saline was intravenously administered to rats for 2 h to exert a water load, and 1 h after the subsequent administration of VE, IM, or dDAVP, sample kidneys were harvested. AQP2 was distributed in the cytoplasm of the collecting duct cells of the cortex in the VE-treated rats (**A**). In the rats treated with IM, AQP2 was more accumulated on the luminal side than the cytoplasm of the collecting duct cells (**B**). dDAVP caused the AQP2 molecule to localize to the luminal side of the collecting duct cells (**C**).

After saline was administered to rats, the AQP2 accumulation was distributed in the cytoplasm of collecting duct cells (Fig. 7A). We observed that AQP2 accumulated on the luminal side of the collecting duct in the rats treated with IM compared to the distribution in the cytoplasm after water loading (Fig. 7B). When dDAVP was administered to rats after saline loading, AQP2 molecules migrated to the luminal plasma membrane of collecting duct cells (Fig. 7C).

#### Urine AQP2 excretion

**Figure 8:**
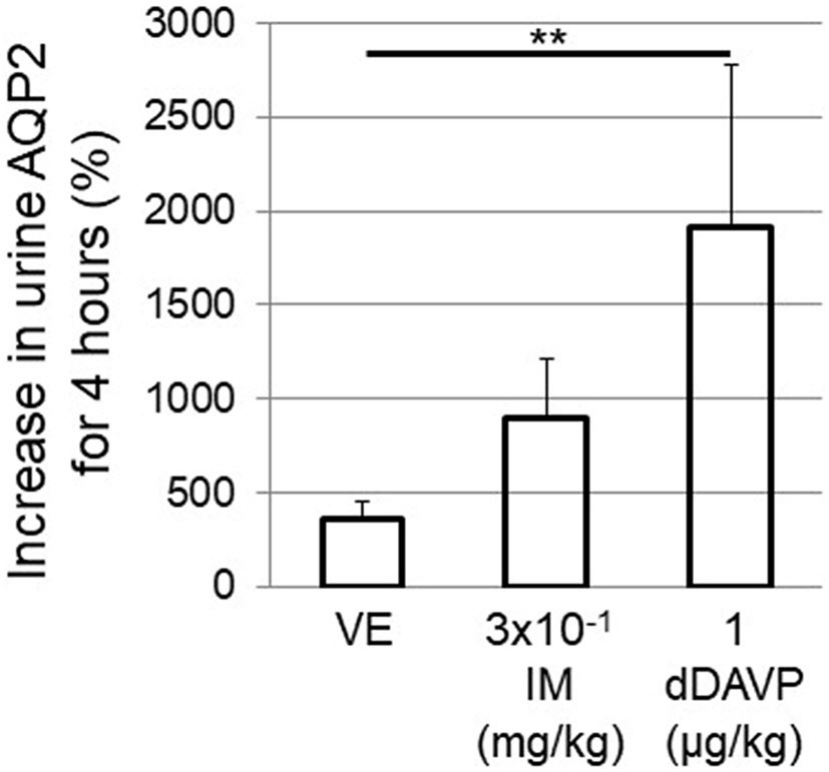
The percentage increase in the release of urine AQP2 from the kidney was compared among intravenous VE, IM, and dDAVP administrations. Values are the mean ± SEM of the measurements. *p < 0.05, **p < 0.01 between values.

The rate of increase in urinary AQP2 excretion was determined and compared with the value before the administration of VE, IM, or dDAVP. Compared to the VE administration, the dDAVP treatment clearly resulted in an increase in urinary AQP2 excretion (Fig. 8). Although the IM administration increased the urinary AQP2 excretion, the increase was not significant.

## Discussion

The results of this study demonstrated that water was absorbed from the rat bladder but was not influenced by the administration of any of the three anticholinergics used. In rats that were diuretic with saline, the use of anticholinergics resulted in the renal reabsorption of water in the absence of AVP. This is the first report that anticholinergics increased the cAMP level and that AQP2 was trafficked to the luminal side of the collecting ducts of the kidney.

Several animal studies have shown that water moves from the bladder epithelium to the systemic circulation^8,12–14^. To date, among the 11 isoforms of AQP observed in mammals, the expressions of AQP2, 3, 4, 7, 9, and 11 have been observed in the bladder epithelium. However, the function of AQP is not fully elucidated^15^. In an earlier investigation, when physiological saline was injected into the bladder, water was absorbed from the bladder epithelium with an enhancement of epithelial AQP2 expression^8^. The bladder epithelium has a role in sodium transport, possibly via epithelial sodium channels (ENaCs) or claudin-3,6^16,17^. In humans, > 100 ml of bladder urine disappears during sleep at night, when the bladder capacity reaches its functional limit as shown by the time-course monitoring of bladder capacity by transabdominal 3D ultrasound^18^.

Our present findings in an animal model confirmed that physiological saline and glucose solution injected into the rat bladder were each absorbed through the bladder. The lower the osmotic pressure of the solution administered into the bladder, the higher the concentration effect was, but there was no significant difference. The administration of anticholinergics did not affect the absorption rate. Electrolytes such as Na^+^ and Cl^−^ were also absorbed with water. Changes in the electrolytes, osmotic pressure, and pH of the solutions were observed when the values obtained before and after the solutions' injection were compared; however, no significant differences were observed in these changes even when the anticholinergics were administered. Therefore, although urine absorption from the urothelium of the bladder is possible, the absorption rate was approx. 10% of the injected amount. The urinary absorption mechanism through the bladder epithelium was not activated by the present administration of anticholinergics. These results suggest that urine production may be suppressed by anticholinergics via an increase in the reabsorption of urine in the kidneys.

With the use of animal diuretic models Watanabe et al. demonstrated that anticholinergics have antidiuretic effects^5^. Yamazaki et al. also reported that imidafenacin might enhance the vasopressin-signaling pathway in rats, they did not show that the point of action was the kidney^19^. The present study's results demonstrate for the first time that the antidiuretic effect of anticholinergics is to promote renal urine reabsorption. However, the question thus arises: why do anticholinergics promote urine reabsorption in the kidneys?

Activation of mAChRs in the brain promotes the release of AVP^20,21^. Anatomical evidences have shown that subfornical organ and median preoptic nucleus of the hypothalamus project cholinergic nerve terminals to the paraventricular nuclei and supraoptic nuclei, and activate AVP neurons via mAChRs^22,23^. Especially, M2mAChR promotes AVP synthesis in the supraoptic nuclei^24^. In the present study, however, IM, which has low intracerebral transferability, was used for intravenous administration, and previous experiments confirmed that IM administration did not affect plasma AVP level^5^. Therefore, it is unlikely that anticholinergics acted on the brain to promote the release of AVP.

Cholineacetyltransferase (ChAT) mRNA is localized within the renal cortex collecting ducts, and ChAT-positive cells correspond to the principal cells in the collecting ducts^25^. Endogenous ACh has been suggested to be synthesized in renal cortical cells in rabbits^26^. An increase in the interstitial sodium concentration stimulated endogenous ACh release in the renal cortex^26,27^. It was also reported that the administration of the Na^+^/K^+^-ATPase inhibitor ouabain produced a release of ACh and that the ENaC inhibitor benzamil suppressed the release of ACh^27^. It thus appears that ACh release is associated with sodium ion transport in the renal cortex.

On the other hand, ACh causes Na^+^ diuresis in the collecting ducts in accord with the intracellular Na^+^ concentration^19,23–25^. Takeda et al. reported that ACh suppressed the inward current of ENaCs in the collecting ducts of the rabbit renal cortex, inducing natriuresis^28^. Garg et al. also demonstrated that the muscarinic receptor agonist carbachol had an inhibitory effect on Na^+^/K^+^-ATPase activity in Madin-Darby canine kidney cells via the activation of PKC^29^. The acetylcholinesterase inhibitor distigmine activated endogenous ACh and caused an increase in urine output in a study by Yamazaki et al.^19^ and Williams et al. observed that exogenous ACh increased sodium excretion in the urine^30^.

In the present experiment in which saline loading caused Na^+^ diuresis, the ACh released from the renal cortex may have resulted in a tendency toward natriuresis in an autocrine or paracrine fashion. From this point of view, anticholinergics have the potential to decrease the production of urine through a reabsorption of sodium across the collecting duct cells. In fact, we observed that the high dose of the anticholinergic IM significantly reduced the excretion of urinary sodium, suggesting that sodium reabsorption occurred in the rat kidney. In addition, since the cAMP level was increased by the administration of IM in this study, we speculate that the trafficking of ENaCs to the apical cell membrane occurred, followed by the decrease in the excretion of Na^+^ in the urine. Increased cAMP in the collecting duct cells resulted in the trafficking of ENaCs as well as AQP2 to the apical cell membrane^31^.

Under "steady-state" conditions in normally hydrated animals, the majority of AQP2 is located in the apical plasma membrane^32^. In microdissected rat terminal inner medullary collecting ducts, carbachol inhibited water permeability by activating the phosphoinositide signaling pathway to increase intracellular calcium and activate PKC, independently of the cAMP-mediated hydro-osmotic effect of AVP^7^. It was later clarified that the activation of PKC promoted the internalization of AQP2, and the internalized AQP2 was directed to intracellular vesicles by endocytosis^33,34^. Therefore, endogenous ACh works on diuresis by promoting a PKC-mediated internalization of AQP2 regardless of the level of AVP.

Based on the results of our present experiments, we speculate that the three anticholinergics exerted an antidiuretic effect by inhibiting the internalization of AQP2 in the renal cortex collecting ducts. In fact, we observed the immunofluorescent localization of AQP2 on the luminal side of the cortex collecting ducts. It is likely that anticholinergics will be effective for the reabsorption of urine when endogenous ACh causes diuresis. This study is the first to reveal that anticholinergics showed trafficking of AQP2 on the luminal side. However, it is not yet known why this trafficking is accompanied by an increase in cAMP.

mAChRs are expressed in the kidney glomeruli, proximal tubules, and collecting ducts^6^. The presence of mAChRs in the kidney was confirmed in rabbits, and both principal cells and intercalated cells of the collecting ducts showed M1mAChR, with stronger reactivity in medullary cells than in cortical cells^35^. M4- and M5mAChRs were also present in intercalated-like cells^6^. The M2- and M4mAChRs are coupled to G protein (G_i/o_) to inhibit stimulated adenylate cyclase and reduce intracellular cAMP levels, while M1- and M3mAChRs are coupled to the phosphoinositide pathway through G_q/11_^36^. In addition, M1- and M3mAChRs have been suggested to modulate cAMP production in reconstituted systems in transfected cell lines^37,38^.

In the present study, the level of cAMP before the saline administration (the non-diuretic state) was the same as that in the diuretic state, suggesting that endogenous ACh does not affect the production of cAMP. We speculate that cAMP will be produced when the M2- or M4mACh-mediated inhibitory influences on adenylate cyclase are released by the administration of an anticholinergic. In fact, IM has a high affinity for M1 and M3 receptors, but it also has an affinity for M4 receptors^39^. AT and TO have no muscarinic receptor selectivity. We observed a significant increase in cAMP production following the IM administration, inducing the enhancement of AQP2 trafficking to the apical cell membrane. The underlying mechanism of AQP2 trafficking will be elucidated in more detail in future experiments.

The urinary release of AQP2 is influenced by the action of AVP in the kidney collecting ducts^40^. An increase in the urinary release of AQP2 has been reported in patients with SIADH (syndrome of inappropriate secretion of anti-diuretic hormone), cognitive heart failure, or hepatic cirrhosis, and in pregnant women, while a decrease in the urinary release of AQP2 was reported in patients with chronic kidney diseases (including polycystic kidney) and patients treated with a V2 receptor antagonist^40–42^. The urinary release of AQP2 is related to the apical trafficking of AQP2, which indicates that AQP2 in urine is a useful biomarker that reflects the potency of AVP in the renal collecting ducts^42^. In the present study, dDAVP significantly increased the urinary AQP2, while IM administration also increased AQP2 but not significantly. We speculate that the reason why the increase in cAMP/AQP2 by the administration of an anticholinergic is smaller than that produced by treatment with dDAVP is not the direct action of the anticholinergic on cAMP production; rather, it is the effect of blocking the inhibitory system via the ACh receptors.

### Study limitations

An important limitation of the present study is that we did not measure the endogenous ACh release from the renal cortex during saline loading. A microdialysis technique is necessary to measure the ACh release from the renal cortex^27^. We also did not evaluate the trafficking of ENaCs in order to determine the reason for the decrease in urinary Na^+^ with the high dose of anticholinergics. Further studies may reveal the precise effects of anticholinergics on sodium channels.

## Materials and methods

10- to 12-week-old female Sprague–Dawley rats (190–250 g) purchased from Japan SLC (Shizuoka, Japan) were housed at a constant temperature of 23 °C and 50–60% humidity under a regular 12-h light/dark schedule at the University of Fukui Animal Center. Tap water and standard rat chow were freely available. All animal experiments were conducted according to Fukui University’s Animal Care and Use Committee guidelines, and all experimental protocols were approved by the Fukui University Ethics Commission (number R02059). This study was carried out in compliance with the ARRIVE guidelines.

### Drugs

IM was gifted from Kyorin Pharmaceutical Co., Ltd (Tokyo, Japan). IM was dissolved in water with 1 M HCl and then neutralized with 1 M NaOH and then diluted using 0.9% saline (0.9%w/v of NaCl). AT (Sigma-Aldrich Japan, Tokyo, Japan) was dissolved in warm water and TO tartrate (Kemprotec Limited,Cumbria, UK) was dissolved in 0.9% saline. dDAVP (Desmopressin Acetate, FUJIFILM Wako Pure Chemical Co., Osaka, Japan) was dissolved in water and then diluted using 0.9% saline. VE was 0.9% saline (0.9%w/v of NaCl).

### Reabsorption in the bladder

Under urethane anesthesia, both ureters of rats were ligated, and a catheter was placed in the bladder transurethrally. While the bladder pressure was measured simultaneously, 0.3% saline, 0.9% saline, 5% glucose solution, 10% glucose solution, or soybean oil was injected (n = 8 rats each) until the bladder pressure reached 30 cmH_2_O. The bladder pressure was then maintained at 30 cmH_2_O for 2 h, and then the amount of saline, glucose solution, or oil in the bladder was measured.

We defined the absorption rate as the value obtained by dividing the difference between the amount of saline, glucose solution, or oil injected into the bladder and the amount collected 2 h later. We also compared the absorption rate between when the bladder pressure was maintained at 20 cmH_2_O for 2 h and when it was maintained at 30 cmH_2_O for 2 h (n = 8 rats each). The absorption rates were also measured when the bladder pressure was maintained at 20 cmH_2_O for 1, 2, and 3 h (n = 8 rats each).

The changes in electrolyte concentration and osmolality of the injected solution were evaluated after 0.3% or 0.9% saline was injected into the bladder and the bladder pressure was maintained at 30 cmH_2_O for 2 hr. Sodium and chlor concentration in the solution were measured by the ion-selective electrode method using various electrolyte measuring reagents (Sekisui medical, Co., Tokyo, Japan).

We also investigated whether bladder absorption changes when an anticholinergic was administered intravenously. At 1 min after the intravesical pressure reached 30 cmH_2_O, vehicle (VE) or an anticholinergic, i.e., IM (0.1 mg/kg), TO (0.3 mg/kg), or AT (0.1 mg/kg) was intravenously administered (n = 8 rats each). The changes in electrolytes and osmolality of the injected solution were evaluated before and after the administration of the anticholinergics.

### Reabsorption in the kidney

#### Measurement of the urine amounts

Under urethane anesthesia, bilateral ureters were each cannulated with a small-dia. catheter (SP-10 catheter, inside dia. 0.28 mm, outside dia. 0.61 mm, Natsume Seisakusho, Tokyo). The total amount of urine collected from the two catheters was used as the kidney-derived urine amount. Physiological saline was continuously administered intravenously to obtain a diuretic state. In the first hour, physiological saline was intravenously administered at the rate of 5 ml/h, and then continuously administered at 3 ml/h for 5 h. At 2 h after the start of the saline administration, VE, an anticholinergic (IM; 10^−3^ to 3 × 10^−1^ mg/kg, TO; 3 × 10^−1^ mg/kg, or AT; 10^−1^ to 1 mg/kg), or dDAVP; 1 µg/kg were intravenously administered. The amount of urine was measured every 2 h.

#### Measurement of sodium excretion

The sodium concentration in the collected urine was measured, and the amount of the excreted sodium was calculated by multiplying the amount of urine for 2 h and its concentration. The sodium excretion for 2 h was measured after the administration of vehicle, anticholinergic (IM, TO, AT).

#### Measurement of intracellular cAMP

The cAMP levels in the control, VE-, IM-, and dDAVP-treated rat kidney cortex or medulla were measured using a Cyclic AMP ELISA Kit (Cayman Chemical, Ann Arbor, MI). First, at 2 h after the start of saline administration, VE, IM, or dDAVP was intravenously injected. One hour later, sample kidneys were harvested. Rat kidney before saline administration (non-diuretic state) was used as the control.

The details of the cAMP assay were as follows. The frozen tissue weighing 30–40 mg was dropped into 5 volumes of 5% trichloroacetic acid (TCA). The sample on ice were homogenized using an Automill (Tokken, Chiba, Japan). The homogenates were centrifuged at 1500 g for 10 min at 4 ºC to remove the precipitate. The supernatant was transferred into a clean test tube, and then the TCA was extracted from the sample using water-saturated ether, discarding the ether layer. The extraction was performed two more times.

The sample with the ether layer removed was heated at 70 ºC for 5 min to eliminate the residual ether from the aqueous layer. The sample was transferred to a mouse anti-rabbit IgG pre-coated plate together with serial dilutions of cAMP for a standard curve in duplicate. cAMP AChE Tracer was added, followed by the cAMP ELISA's antiserum. The plate was incubated overnight (18 h) at 4 ºC with shaking. After washing five times in washing buffer, Ellman's reagent was added to the well and the plate was shaken for 60 min at 37 ºC under cover and in the dark. The plate was then read at a wavelength 412 nm on a Spectra Max M5 (Molecular Devices, Sunnyvale, CA).

#### Immunofluorescence

At 2 h after the start of saline administration, VE, IM (0.3 mg/kg), or dDAVP was intravenously administered to rats (n = 6), and 1 h later, sample kidneys were harvested. Kidney tissues were frozen in liquid nitrogen and sliced into 8-μm sections. Tissue samples on glass slides were fixed with acetone at 4 °C and dried at room temperature for 10 min. After blocking with 1% bovine serum albumin for 1 h, the sections were incubated with a rabbit anti-rat AQP2 antibody (Novus Biologicals, Centennial, CO) at 4 °C overnight. After being washed with phosphate-buffered saline, the sections were incubated with Goat anti-Rabbit IgG(H + L) Cross-Adsorbed Secondary Antibody, Alexa Fluor 488 (Thermo Fisher Scientific, Waltham, MA) for 90 min. The nuclei were stained with DAPI (Thermo Fisher) at room temperature for 15 min. Confocal images were obtained by confocal laser scanning microscopy (FV1200, Olympus, Tokyo).

#### Measurement of urine AQP2 excretion

Urinary AQP2 concentrations were measured by an AQP2 ELISA Kit (Rat) (Aviva System Biology, San Diego, CA) according to the manufacturer's instructions. The detection and capture antibodies were rabbit polyclonal antibodies. Absorbance was measured at 450 nm. The density of yellow coloration read by absorbance at 450 nm was quantitatively proportional to the amount of sample AQP2. The rate of increase in the release of urinary AQP2 from the kidney was calculated with the following formula: the amount of urinary AQP2 released during the 4 h after the start of drug administration (VE, IM, or dDAVP) was subtracted from the amount of urinary AQP2 released during the 2 h before the drug administration, and this was divided by the amount of urinary AQP2 released during the 2 h before the drug administration.

### Statistical analysis

Results are presented as the mean ± standard error of the mean (SEM). The Wilcoxon rank sum test and the unpaired t-test were used to compare the differences between two groups. The statistical differences among more than two groups were analyzed by one-way analysis of variance (ANOVA) followed by Dunnett's multiple comparison test or Tukey's test. A p-value < 0.05 was considered significant.
